# The Association of the Epidermal Growth Factor Receptor (EGFR) Immunoexpression With Prognostic Parameters in Adenocarcinoma Patients Receiving Neoadjuvant Treatment

**DOI:** 10.7759/cureus.56763

**Published:** 2024-03-23

**Authors:** Derya Demir, Murtaza Parvizi, Burcin Pehlivanoglu, Erhan Ergin, Semin Ayhan, Basak Doganavsargil

**Affiliations:** 1 Pathology, Ege University, Izmir, TUR; 2 Radiation Oncology, Manisa City Hospital, Manisa, TUR; 3 Pathology, Dokuz Eylul University, Izmir, TUR; 4 Internal Medicine, Manisa City Hospital, Manisa, TUR; 5 Pathology, Manisa Celal Bayar University, Manisa, TUR

**Keywords:** egfr, adenocarcinoma, prognosis, neoadjuvant radiotherapy, neoadjuvant chemoradiotherapy

## Abstract

The epidermal growth factor receptor (EGFR) expression is considered to play an essential role in the pathogenesis of colorectal adenocarcinoma. This study assessed the expression and predictive/prognostic value of EGFR expression in pre-op biopsy and post-op resection specimens in patients receiving neoadjuvant radiotherapy/neoadjuvant chemoradiotherapy (NRT/NCRT).

Thirty-four consecutive patients were included in this study. The association between the prognostic features and EGFR immunohistochemical expression was analyzed in pre- (n=34) and post-treatment (n=22) tissue samples in cases with available tissue blocks.

Of 34, 23 (67.6%) were men. The median age was 60.50 ± 10.69 (range, 31-84) years. EGFR expression was detected in 88.2% of biopsy specimens and in 91.2% of surgical specimens. There was only slight agreement between pre-op and post-op EGFR expression scores (kappa value 0.11). There was no significant correlation between pre-op and post-op EGFR expression scores (p>0.05). Although pre-op EGFR positivity and higher pre-op EGFR scores seemed to indicate a worse prognosis, this association between pre-op EGFR expression and overall survival (OS) or disease-specific survival (DSS) did not reach statistical significance (p>0.05). The only case with a post-op EGFR score of three who died of the disease experienced local recurrence and had distant metastasis.

In conclusion, EGFR positivity in pre-op biopsy samples seems to be associated with shorter survival, and increased EGFR expression in post-treatment resection specimens predicts aggressive behavior in patients with rectal adenocarcinoma who received NRT/NCRT. However, due to the molecular heterogeneity, EGFR expression status should be evaluated in resection specimens rather than in pre-op biopsy samples for optimal prognosis prediction.

## Introduction

Growth factor receptors are critical for growth, differentiation, and tumor cell motility in carcinogenesis, and epidermal growth factor receptor (EGFR) is the most studied growth factor receptor, regulating cell proliferation, differentiation, apoptosis, angiogenesis, and metastasis [1-3]. Gene amplification and/or protein overexpression of EGFR have been demonstrated in vivo in numerous solid tumors, and its overexpression has been associated with poor prognosis [4-9].

Previously, EGFR overexpression has been found to be associated with shorter survival in patients with rectal adenocarcinoma [10,11]. Colorectal carcinomas are molecularly heterogeneous tumors, and there is an increasing need for biomarkers for targeted therapy. In the treatment of rectal cancer, neoadjuvant radiotherapy/neoadjuvant chemoradiotherapy (NRT/NCRT) is considered to be more advantageous compared to adjuvant radiotherapy (ART) in terms of reducing the tumor burden, increasing the likelihood of sphincter-sparing procedures, and reducing acute and late radiation-associated toxicities since intact intestinal tissues are outside the radiation field [7,12]. However, studies on response to anti-EGFR therapy in rectal cancer show controversial results, although some authors have claimed that EGFR status may predict response to neoadjuvant therapy [13-15].

Therefore, in this study, we aimed to investigate EGFR expression in both pre- and post-treatment tissues and to determine its predictive value in biopsy specimens as well as its association with morphological and clinical prognostic parameters and treatment response in patients receiving NRT/NCRT.

## Materials and methods

Study population

This study was approved by the institutional ethics committee (Reference no: 70198063-050.06.04 - 16-12.1/1). Of 50 consecutive patients with rectal adenocarcinoma who received NRT/NCRT between 2010 and 2016, 34 were included in the study. The association between the prognostic features and EGFR immunohistochemical expression was analyzed in pre- (n=34) and post-treatment (n=22) tissue samples in cases with available tissue blocks, including adequate tumors. Patients receiving adjuvant treatment were excluded from the study. 

Demographic, clinical, and histological prognostic data of the patients, including age, gender, tumor location and size, histological grade of the tumor, preoperative clinical stage, and postoperative pathological Tumour, Node, Metastasis (TNM) stage [16], perineural invasion (PNI) and lymphovascular invasion (LVI) in the surgical specimen, surgical margin status, and tumor regression grade after neoadjuvant therapy were recorded for each patient. Tumor regression grading was performed according to the Mandard classification system, which is commonly used to assess the extent of tumor regression in response to neoadjuvant treatment, particularly in the context of colorectal cancer [17]. In addition, prognostic parameters associated with survival, including disease-specific survival (DSS), overall survival (OS) time, recurrence, metastatic characteristics at the time of diagnosis, in case of recurrence or metastasis, and after NRT/NCRT as well as the RT dose and technique, surgical technique were evaluated.

Immunohistochemical analysis

Sections of 3-5 μm thickness prepared from formalin-fixed paraffin blocks were stained with EGFR primary antibody (clone: ​​EGFR.113, Leica-Novocastra, 1:20 dilution) using a LeicaBondMax (Leica Biosystems, Wetzlar, Germany) automatic staining device. Membranous and/or cytoplasmic staining was accepted as positive. The extent of EGFR staining was scored as follows in both pre-treatment biopsy specimens and post-treatment surgical specimens: 0 (negative), 1 (1-9%), 2 (10-49%), and 3 (≥50% of the tumor).

Statistical analysis

Statistical analysis was performed using SPSS 23.0 for Windows (IBM Inc., Armonk, New York). The normal distribution of data was assessed by the Shapiro-Wilk test. Continuous variables were expressed as mean, standard deviation (SD), or median (interquartile ratio, IQR), and categorical variables were expressed as frequencies (n) and percentages (%). Continuous variables were compared using the Kruskal-Wallis test. Categorical variables were compared using the chi-square test. Survival curves were compared using the log-rank (Mantel-Cox) test for all defined endpoints. A p-value of <0.05 was considered significant.

## Results

Clinical findings

The 34 patients comprised 23 (67.6%) men and 12 (32.4%) women with a median age of 60.50 (range, 31-84) years. The tumor was localized to the lower rectum (1-6 cm from denteta line) in 28 (82.4%) and to the middle rectum (>7 cm) in six (17.6%) patients. The great majority was evaluated as cT3 (n=31, 91.2%), while the remaining three were cT4 (8.8%) pre-operatively. More than two-thirds were found to have nodal metastatic disease on pre-op radiological examination (cN0: 8 (23.5%), cN1: 15 (44.1%), and cN2: 11 (32.4%)).

The NRT dose, depending on the RT technique, was 5000 cGy (intensity-modulated radiotherapy (IMRT)), 4500 cGy primary tumor + primary lymphatics, 5000 cGy primary tumor bed (simultaneously integrated boost (SIB) + set-up margin) in 20 (58.8%) patients and was 4500 cGy (3D conformal radiotherapy (3DCRT)): primary tumor + primary lymphatics + set-up margin) in 14 (41.2%) patients. While 29 (85.3%) patients received two cycles of CT concurrently with RT, the remaining five (14.7%) patients could not receive concomitant CT due to advanced age and comorbidities. Of the 29 patients who could receive neoadjuvant chemotherapy, 18 patients received 5-FU/LV, and 11 received oral capecitabine.

In the postoperative period, one patient received ART, and 19 received adjuvant CT (5-fluorouracil plus l-leucovorin (FOLFOX regime) (n=11) or 5-FU/LV (n=6) or oral capecitabine (n=2)). Of these 19 patients, 16 received six cycles, two patients received four cycles, and one patient received 12 cycles of CT.

Following NRT, low anterior resection was performed in 16 (47.1%), and Miles operation was performed in 16 (29.4%). Surgery could not be performed in eight patients due to refusal of surgery (n=5), comorbidities (n=1), and unresectability (n=2).

Histopathological findings

All 34 cases showed features consistent with adenocarcinoma, two of which were mucinous type. Postoperative histopathological examination following NRT/NCRT was performed in 26 patients (Table 1).

**Table 1:** Histopathological findings in resection specimens *Two cases with residual tumor had mucinous carcinoma.

Differentiation	n (%)*
Well-differentiated	9 (26.5%)
Moderately differentiated	11 (32.4%)
Poorly differentiated	1 (2.9%)
No tumor	3 (8.8%)
Lymphovascular invasion	n (%)
Present	8 (30.7%)
Absent	18 (69.2%)
Perineural invasion	n (%)
Present	1 (3.8%)
Absent	25 (96.2%)
ypT stage	n (%)
ypT0	3 (8.8%)
ypT1	2 (5.9%)
ypT2	8 (23.5%)
ypT3	13 (38.2%)
ypN stage	n (%)
ypN0	17 (50.0%)
ypN1	6 (17.6%)
ypN2	3 (8.8%)
Tumor regression score per Mandard’s classification	n (%)
1 (Complete response, no viable tumor cells)	3 (8.8%)
2 (Single tumor cells or small groups of neoplastic cells)	2 (5.9%)
3 (Residual cancer with predominant fibrosis)	7 (20.6%)
4 (Residual cancer with focal fibrosis)	11 (47.5%)
5 (No response, extensive residual cancer without fibrosis)	1 (2.5%)

Association between EGFR expression scores and prognostic parameters

Immunohistochemical EGFR staining was performed on preoperative biopsy specimens in 34 cases and on postoperative surgical specimens in 22 cases. EGFR expression was detected in 88.2% of biopsy specimens and in 91.2% of surgical specimens. Overall, there was only slight agreement between the pre-op and post-op EGFR expression scores (kappa value 0.11) (Table 2). Also, there was no significant correlation between the pre-op and post-op EGFR expression scores (p>0.05). Pre-op EGFR score was not statistically associated with age, gender, tumor size, location, tumor regression grade (Mandard score), cT stage, cN, or cM stage (p>0.05). Similarly, postoperative EGFR expression score was not significantly associated with age, gender, tumor location, pT stage, pN stage, tumor regression grade (Mandard score), PNI, or LVI (p>0.05 for all). Tumors with post-op EGFR positivity (n=18) were larger than the EGFR negative (n=3) tumors; however, the difference did not reach statistical significance (2.85 ± 1.08 cm vs. 2.0 ± 1.52 cm, p=0.073). The only case with a post-op EGFR score of 3 experienced local recurrence and had distant metastasis.

**Table 2:** Comparison of pre- and postoperative EGFR expression scores Measure of agreement Kappa: 0.11 (slight agreement) 0 negative, 1 positive (1-9%), 2 positive (10-49%), and 3 positive (≥50% of the tumor) EGFR - epidermal growth factor receptor

		Post-op EGFR score				Total
		0	1	2	3	
Pre-op EGFR score	0	0	1	2	0	3
	1	0	2	1	0	3
	2	1	3	5	0	9
	3	2	1	3	1	7
Total		3	7	11	1	22

The type and/or technique of the neoadjuvant treatment did not have a significant effect on post-op EGFR expression (p>0.05).

Association between EGFR expression scores and survival

Local recurrence was observed in two (6.1%), and distant metastasis was detected in six (18.2%) patients (diffuse metastatic disease in three, liver metastasis in two, lung metastasis in one, and brain metastasis in one patient) during the follow-up period. One patient who died in the perioperative period was excluded from the survival analysis.

Mortality occurred in nine (27.7%) patients, five of whom died of the disease. Median survival was 78 months (45-N/A, 95% CI). one-, three- and five-year overall survival (OS) was 97% (91.3%-100%, 95% CI), 77% (62.5%-95.9%, 95% CI) and 65% (46.9%-89.9%, 95% CI), respectively, while one-, three-, and five-year DSS was 97% (91.3%-100%, 95% CI), 87.2% (73.9%-100%, 95% CI) and 80.4% (64%-100%, 95% CI), respectively.

**Figure 1:**
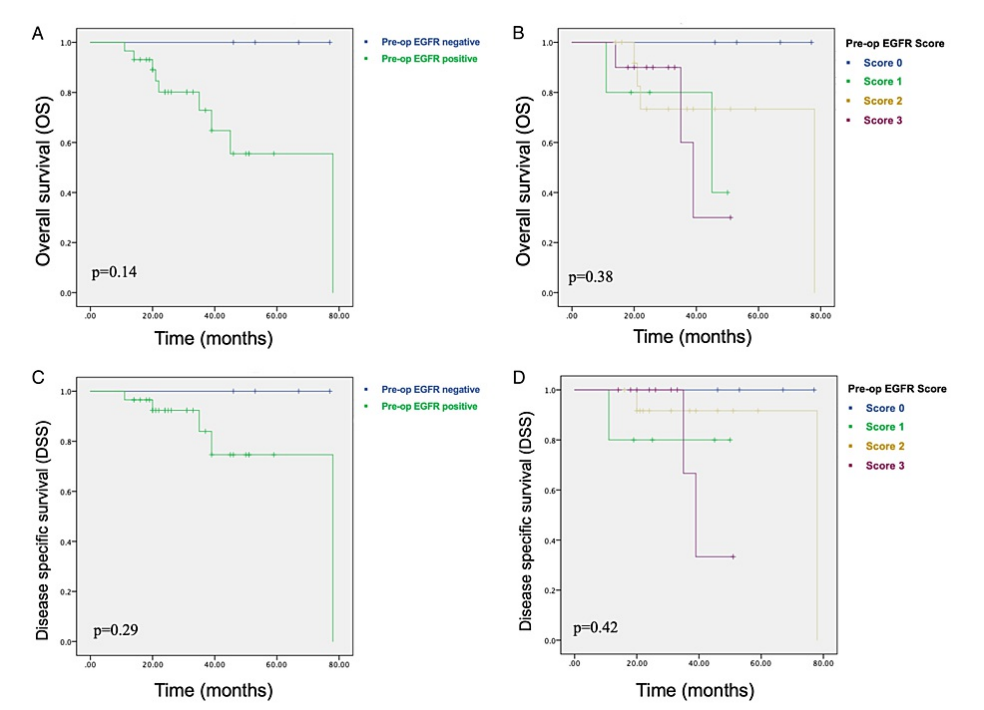
Association between pre-op EGFR expression and survival A) Pre-op EGFR positive-negative groups and overall survival: Kaplan-Meier analysis; B) Pre-op EGFR score and overall survival: investigating the differences; C) Pre-op EGFR positive-negative groups and disease-free survival: Kaplan-Meier analysis; D) Pre-op EGFR score and disease-free survival: assessing the impact on outcomes. EGFR - epidermal growth factor receptor

**Figure 2:**
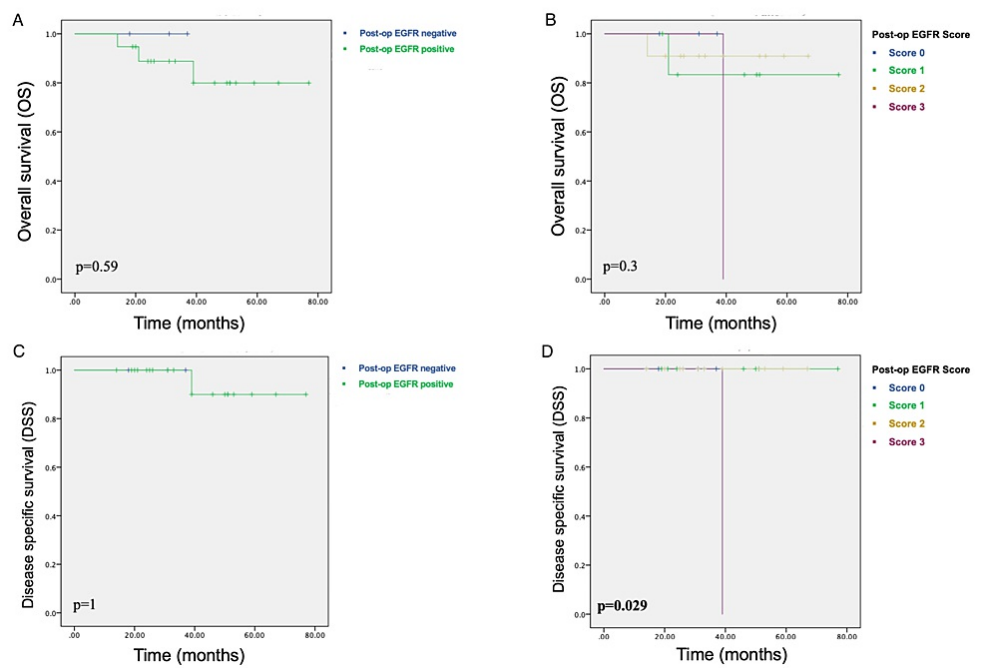
Association between post-op EGFR expression and survival A) Post-op EGFR positive-negative groups and overall survival: Kaplan-Meier analysis; B) Post-op EGFR score and overall survival: investigating the differences; C) Post-op EGFR positive-negative groups and disease-free survival: Kaplan-Meier analysis; D) Post-op EGFR score and disease-free survival: score three is significantly associated with a worse prognosis. EGFR - epidermal growth factor receptor

Although pre-op EGFR positivity and higher pre-op EGFR scores seemed to indicate a worse prognosis, this association between pre-op EGFR expression and OS or DSS did not reach statistical significance (p>0.05) (Figure 1, Table 3). Among 22 cases with available resection specimens, only one with an EGFR score of three died of the disease (Figure 2, Table 4).

**Table 3:** Association between pre-op EGFR expression and survival EGFR - epidermal growth factor receptor

Pre-op EGFR status	n	Overall survival (OS)						Disease-specific survival (DSS)					
		n of diseased cases	Mean (months)	SD (months)	Median (months)	95% confidence interval	p-value	n of diseased cases	Mean (months)	SD (months)	Median (months)	95% confidence interval	p-value
No EGFR expression	4	0	78	0	N/A	N/A	p=0.14	0	78	0	N/A	N/A	p=0.29
EGFR expression present	29	9	56.8	5.96	78	39.0-N/A		5	66	5.41	78	N/A	
EGFR score 0/1	9	2	65.7	7.84	NaN	45.0-N/A	p=0.55	1	70.6	7.02	N/A	N/A	p=0.67
EGFR score 2/3	24	7	58.7	6.49	78	39.0-N/A		4	65.9	6.2	78	39.0-N/A	
EGFR score 0	4	0	78	0	N/A	N/A	p=0.38	0	78	0	N/A	N/A	p=0.42
EGFR score 1	5	2	51.4	12.99	45	45.0-N/A		1	64.6	11.99	N/A	N/A	
EGFR score 2	14	4	62.8	7.54	78	N/A		2	73.2	4.63	78	N/A	
EGFR score 3	10	3	47	10.66	39	35.0-N/A		2	50.7	11.2	39	35.0-N/A	

**Table 4:** Association between postoperative EGFR expression and survival EGFR - epidermal growth factor receptor

Post-op EGFR status	n	Overall survival (OS)						Disease-specific survival (DSS)					
		n of diseased cases	Mean (months)	SD (months)	Median (months)	95% confidence interval	p-value	n of diseased cases	Mean (months)	SD (months)	Median (months)	95% confidence interval	p-value
No EGFR expression	3	0	77	0	N/A	N/A	p=0.59	0	77	0	N/A	N/A	p=1
EGFR expression present	19	3	67	5.24	N/A	N/A		1	73.2	3.6	N/A	N/A	
EGFR score 0/1	10	1	70	6.55	N/A	N/A	p=0.73	0	77	0	N/A	N/A	p=0.41
EGFR score 2/3	12	2	65.9	6.97	N/A	N/A		1	70.7	5.78	N/A	N/A	
EGFR score 0	3	0	77	0	N/A	N/A	p=0.3	0	77	0	N/A	N/A	p=0.029
EGFR score 1	7	1	67.7	8.52	N/A	N/A		0	77	0	N/A	N/A	
EGFR score 2	11	1	71.3	5.46	N/A	N/A		0	77	0	N/A	N/A	
EGFR score 3	1	1	39	0	39	N/A		1	39	0	39	N/A	

Mandard score was significantly associated with OS (p<0.0001). While Mandard 1-3 cases had similar mean OS (77 ± 0 months), Mandard 5 cases had significantly shorter OS compared to Mandard 1-3 and Mandard 4 cases (14 ± 0 vs. 65.1± 7.31 vs. 77 ± 0 months, p=0.073 for Mandard 5 vs. Mandard 3, and p=0.009 for Mandard 5 vs. Mandard 4). However, no statistically significant association was found between the Mandard score and DSS (p=0.96).

Patients with local recurrent disease had significantly shorter median OS (29.5 ± 6.72 months vs. 78 ± 4.94 months, p=0.013). DSS was significantly shorter in cases with distant metastasis (mean 49.8 ± 11.1 vs. 75.5 ± 2.47, p=0.007) and local recurrence (median 29.5 ± 6.72 months vs. 78 ± 3.51 months, p=0.00071). No other significant association was found regarding OS or DSS.

## Discussion

In this study, we evaluated EGFR expression in pre-treatment biopsy and post-treatment surgical specimens using immunohistochemistry in patients with rectal adenocarcinoma who received neoadjuvant radio- and/or chemotherapy (NRT/NRCT). While EGFR overexpression has previously been reported in 25-77% of colorectal carcinomas, we detected EGFR expression in 88.2% of biopsy specimens and in 91.2% of surgical specimens [8, 15, 18]. This higher frequency of EGFR positivity than previously reported data may be attributed to populational differences. More strikingly, we observed only slight agreement between the pre-op and post-op EGFR expression scores (kappa value 0.11). This is most likely due to the heterogeneous molecular background of colorectal carcinomas, suggesting that EGFR expression status should be evaluated in resection specimens rather than in pre-op biopsy samples to obtain optimal results [8,15]. 

We did not observe any significant association between histopathological prognostic parameters and pre-op or post-op EGFR expression status, probably due to the small number of patients in the study group. However, pre-op EGFR positivity and higher (≥2) pre-op EGFR scores were associated with shorter survival, albeit without statistical significance (p>0.05), supporting the previous studies which demonstrated that EGFR expression is associated with worse prognosis in patients receiving NCRT for locally advanced rectal cancer [8, 19]. Moreover, among 22 cases with available resection specimens, the only case with an EGFR score of three died of the disease at the 39th month, while the mean survival for the other groups (EGFR score 0, 1, and 2) was 77 months (p=0.029). This significant association suggests that post-op EGFR expression score may be a candidate prognostic factor for rectal adenocarcinomas; however, this patient also had local recurrence and was an advanced-stage patient with distant metastasis; therefore, the effect of EGFR expression on disease-specific survival may be a contributing factor rather than being an independent prognostic factor. This aspect deserves further investigation in a larger series. Of note, this patient was the only patient with elevated post-NRCT CA19-9 level, and this aspect may be investigated in further studies.

The Mandard tumor regression grade, commonly known as the Mandard score, is a histopathological assessment tool used to evaluate the response of solid tumors to neoadjuvant treatment, particularly in the context of gastrointestinal cancers such as esophageal and rectal cancers. This scoring system ranges from one (complete response) to five (no response). The Mandard score provides a standardized method to quantify the histological response of tumors to preoperative therapy, enabling clinicians and researchers to categorize patient outcomes systematically. In this study, no significant association between pre-op EGFR expression scores and Mandard score (tumor regression grade) was found, in contrast to Motlagh et al.’s findings, which have suggested that EGFR status may predict response to neoadjuvant therapy [15]. Also, there was no significant correlation between the pre-op and post-op EGFR expression scores, and no significant association between the type and/or technique of the neoadjuvant treatment and post-op EGFR expression was found. These findings indicate that EGFR expression status may not be a reliable factor to have an impact on neoadjuvant and/or adjuvant treatment options until more evidence can be gathered. In fact, in recent studies, even the efficacy of anti-EGFR therapy has been demonstrated to differ depending on tumor location because of the molecular heterogeneity of colorectal carcinomas [13, 14]. Mandard score, which was significantly associated with OS, as well as local recurrence and distant metastasis, the statistically significant indicators of DSS, remain to be the most crucial factors in choosing adjuvant therapy options. The association with these clinical outcomes emphasizes its utility in tailoring adjuvant treatment plans to improve patient prognosis.

In our study, we used immunohistochemistry, which is an easily applicable method in many laboratories, to assess EGFR expression in rectal adenocarcinoma cases who have received NRT/NCRT. Although there is ongoing research on the methods to be used for the evaluation of protein expression of EGFR in tissue samples, immunohistochemical examination has been shown to be an effective technique [8, 15, 20] and has been shown to be compatible with florescent in situ hybridization [21-23]. However, the optimal immunohistochemical scoring system has not yet been defined [2]. We think that a standard scoring system should be adopted for a more objective evaluation of the association between EGFR status and prognostic markers and to decide the optimal targeted therapy option in patients with colorectal cancer.

The small number of patients is the most important limitation of the current study. However, we think that our study provides unique findings to the literature, and we discussed some concerns regarding the value of EGFR expression in patient management. 

## Conclusions

In conclusion, EGFR positivity in pre-op biopsy samples and in post-treatment resection seems to be associated with shorter survival and aggressive behavior, respectively. However, EGFR expression status may not be a reliable factor to have an impact on neoadjuvant and/or adjuvant treatment options and should preferentially be evaluated in resection specimens for optimal prognosis prediction due to the molecular heterogeneity.
